# Implications of cyclophosphamide, methotrexate, and 5-fluorouracil chemotherapy on hippocampal-dependent cognition and gut microbiome

**DOI:** 10.3389/frmbi.2025.1486757

**Published:** 2025-10-06

**Authors:** Christa Corley, Chase Swinton, Taylor McElory, Bhavana Sridharan, Justin Thomas, Bailey Cronin, Vijayalakshmi Sridharan, Michael S. Robeson, Antiño R. Allen

**Affiliations:** ^1^ Division of Radiation Health, University of Arkansas for Medical Sciences, Little Rock, AR, United States; ^2^ Department of Pharmaceutical Sciences, University of Arkansas for Medical Sciences, Little Rock, AR, United States; ^3^ Department of Bioinformatics, University of Arkansas for Medical Sciences, Little Rock, AR, United States; ^4^ Department of Neurobiology and Developmental Sciences, University of Arkansas for Medical Sciences, Little Rock, AR, United States

**Keywords:** hippocampal, cognitive, cyclophosphamide, methotrexate, 5-fluorouracil

## Abstract

Chemotherapy-induced cognitive impairment, also called “chemobrain”, has been heavily researched as a major side effect of cancer treatment. Although breast cancer has a 91% survival rate in the United States, this rate is significantly lower in developing countries. Cancer survivors often experience chemobrain, which can decrease their quality of life post-chemotherapy. The presented study evaluates potential mechanisms for long-term symptoms in cyclophosphamide, methotrexate, and 5-fluorouracil (CMF)-induced cognitive impairments and implications of CMF on the microbiome. Twelve-week-old C57/B6J female mice were treated with a combination of CMF once a week for 4 weeks. Spatial memory was tested with the Morris water maze. Hippocampal tissues were used to probe for immediate-early genes (IEGs) with Western blotting techniques. Fecal matter was collected to assess microbial community composition via 16S rRNA gene sequencing. In this study, we showed that chemotherapy impaired spatial memory during the Morris water maze trials and resulted in a significant decrease in IEGs c-Fos, Arc, and Zif286 expression. Comparing alpha-diversity, there were no significant differences identified among taxa within the CMF group compared to the saline group for Pielou’s evenness. However, beta-diversity qualitative metrics, Jaccard and Unweighted UniFrac, were significantly different. These results suggest that continual memory deficits may be associated with alterations in synaptic plasticity and long-term potentiation.

## 1 Introduction

With an estimated 297,790 new cases in 2023, breast cancer is the most commonly diagnosed cancer worldwide and in women to date (Arnold et al., 2022). In the United States, the 5-year survival rate of breast cancer is 91%; however, in low- and middle-income countries (LMICs), as defined by the World Bank based on a country’s gross national income bracket (Lencucha and Neupane, 2022), this rate is significantly lower. Countries such as Samoa, Gambia, Fiji, and Brazil have survivorship rates ranging from as low as 25% to 60% (Rivera-Franco and Leon-Rodriguez, 2018; Lei et al., 2021). Nevertheless, the implementation of diagnostics, adjuvant chemotherapy, and further technological advancements have propagated the survival rates of breast cancer globally.

Patients with cancer frequently report cognitive problems that can affect their quality of life. Studies have shown that patients experience cognitive dysfunction during and up to 20 years after treatment (Koppelmans et al., 2012). As breast cancer survivorship rates increase, the need to investigate the short- and long-term effects of breast cancer chemotherapy has become increasingly more prevalent. Cognitive impairments are one of the most common side effects of chemotherapy (Henderson et al., 2019). Also referred to as “chemobrain,” chemotherapy-induced cognitive impairments pose significant functionality challenges for breast cancer survivors. Patients experiencing chemobrain report deficits in memory retention, learning, executive function, and processing speed (Yang et al., 2010; Koppelmans et al., 2012; Brown et al., 2021).

The first efficacious chemotherapy regimen for breast cancer was developed in the 1970s: a polytherapy combining cyclophosphamide, methotrexate, and 5-fluorouracil (CMF). Cyclophosphamide (CYP) is a cytotoxic, alkylating antineoplastic drug that is commonly used as an immunosuppressive (Yang et al., 2010). Synthesized in 1958, CYP was the eighth cytotoxic anticancer drug approved by the Food and Drug Administration (Li et al., 2022). The alkylating nature of CYP has been found to create alkyl crosslinks between DNA, inducing apoptosis (Matalon et al., 2004). CYP has been shown to permeate the blood–brain barrier (BBB), although the underlying mechanisms remain understudied [11]. Methotrexate (MTX), developed in 1949, is an antifolate that inhibits antioxidant activity by binding to dihydrofolate reductase (Skubisz and Tong, 2012; Taran et al., 2023). This consequently hinders the amelioration of oxidative stress (Hess and Khasawneh, 2015), leading to high levels of cytotoxicity in clinical applications (Sekeres et al., 2021). Only at high doses (i.e., 1–3 g/m^2^) has MTX been shown to cross the BBB (Angelov et al., 2009; Prodduturi and Bierman, 2012). 5-Fluorouracil (5-FU) is an anti-metabolite that interferes with thymidine synthesis and DNA replication. Discovered in 1957 (Heidelberger et al., 1957), 5-FU is part of the first class of thymidylate synthase inhibitors used in clinical application (Chu et al., 2003). Designed as an analog of RNA nucleotide base uracil, 5-FU inhibits thymidylate synthase, which consequently disrupts proper DNA formation and instigates cytotoxicity (Wigmore et al., 2010). Similar to CYP, 5-FU has also been shown to penetrate the BBB via passive diffusion (Wigmore et al., 2010). The combination of CYP, MTX, and 5-FU modeled in 1973 by Bonadonna et al (Bonadonna et al., 1995). created a regimen that was instrumental for implementing chemotherapeutic treatments and developing safer surgical procedures for patients with breast cancer (Verrill, 2009).

As the use of CMF chemotherapy to treat breast cancer became more pervasive, the need to study its long-term effects became more pressing. Wieneke and Dienst found that women treated with adjuvant chemotherapy, primarily CMF, displayed mild cognitive impairment compared to test norms (Wieneke and Dienst, 1995). Later work by Schagen et al. revealed that patients treated with CMF and tamoxifen reported significant impairments in concentration, memory, and locomotion compared to control (Schagen et al., 1999). During this time, however, the use of taxanes in combination with anthracyclines (ACs) became the standard for breast cancer polytherapy in the United States (Brown et al., 2021). The introduction of AC regimens denoted a shift away from using CMF moving into the 21st century.

In low- and middle-income countries (LMICs), access to chemotherapeutic treatments for breast cancer remains limited (Sandelin et al., 2002). Newer taxane and AC agents are not widely available in these countries; therefore, the use of CMF remains highly prevalent globally (Tfayli et al., 2010). Furthermore, most chemobrain studies involving patients are conducted in the United States, Europe, Canada, and Australia—all high-income countries (Ribi, 2012). LMICs, particularly those in Africa, such as Uganda, South Africa, and Tanzania, still use the CMF to treat breast cancer (Serra et al., 2020; Menon et al., 2021; Keetile et al., 2023). The urgency to understand the cytotoxic effects of this regimen persists. A study in Gauteng, South Africa conducted in 2021 found that 33% of Black African women (*n*=10) treated with CMF reported significant cognitive impairment (Keetile et al., 2021). Another South African study in 2023 revealed that 77.4% of Black African women (*n*=53) diagnosed with stage II or III breast cancer treated with CMF displayed significant cognitive impairment (Keetile et al., 2023). These studies demonstrate the necessity of continual investigation of how CMF dysregulates brain functionality and how to mitigate the physiological symptoms harming patients with breast cancer and survivors.

Chemotherapy is also known to affect the microbiome–gut–brain (MGB) axis. The gut microbiota–immune–brain axis is becoming increasingly important for the treatment of a myriad of neurological and cognitive disorders (O’Riordan et al., 2025). Studies have found that chemotherapy alters microbiota populations, activates neuroimmune responses, and promotes glial dysregulation (Song and Bai, 2021; Rowaiye et al., 2024). For example, it is well known that the gut microbiome can enhance or mitigate the effectiveness of therapeutic treatments of patients with cancer (Viaud et al., 2013; Alexander et al., 2017). For example, a variety of microbial taxa are important mediators of immune checkpoint inhibitors, while others promote increased toxicity of anticancer drugs, as reviewed by Huange et al (Huang et al., 2022). Furthermore, several studies have shown various levels of responses by the gut microbiota in patients with breast cancer (Wu et al., 2020; Aarnoutse et al., 2022). While the underlying connection between the MGB and chemobrain is poorly understood, some studies have suggested that gut microbiota assists in regulating hippocampal-dependent cognition (Kuijer and Steenbergen, 2023). Furthermore, it is also possible that the MGB contributes to the development of chemobrain, overall fatigue, and other chemotherapy-induced symptoms (Grant et al., 2021; Xiao et al., 2021). For example, MTX influences MGB composition and can engender both gut dysbiosis (Chen et al., 2024; Rowaiye et al., 2024) and proteomic alterations (Letertre et al., 2020).

This study aims to investigate how combination CMF chemotherapy affects hippocampal-dependent behavior and gut-microbiome diversity. This work may provide further insight into possible underlying mechanisms of long-term deficits in hippocampal-dependent functioning due to CMF and the role of the MGB in chemobrain.

## 2 Materials and methods

### 2.1 Animals

After adaptive feeding for 1 week, 12-week-old C57/B6J female mice (The Jackson Laboratory) were randomly assigned to one of the two groups: control (administered saline) or CMF-treated group, with 12 mice per group. The mice were housed (4/cage) in a climate-controlled environment with a constant 12-h light:12-h dark cycle for 30 days. The humidity was maintained between 30% and 60%, with a temperature set point of 72°F, a 12-h light–dark cycle, and ventilation providing 10–15 air changes per hour. Food and water were provided *ad libitum*. Food consumption and weight were recorded throughout the study. Animals were weighed weekly throughout the study to monitor health status and track potential treatment-related changes in body weight. This study was approved by the Institutional Animal Care and Use Committee at the University of Arkansas for Medical Sciences.

### 2.2 Drug paradigm

**Figure 1:**
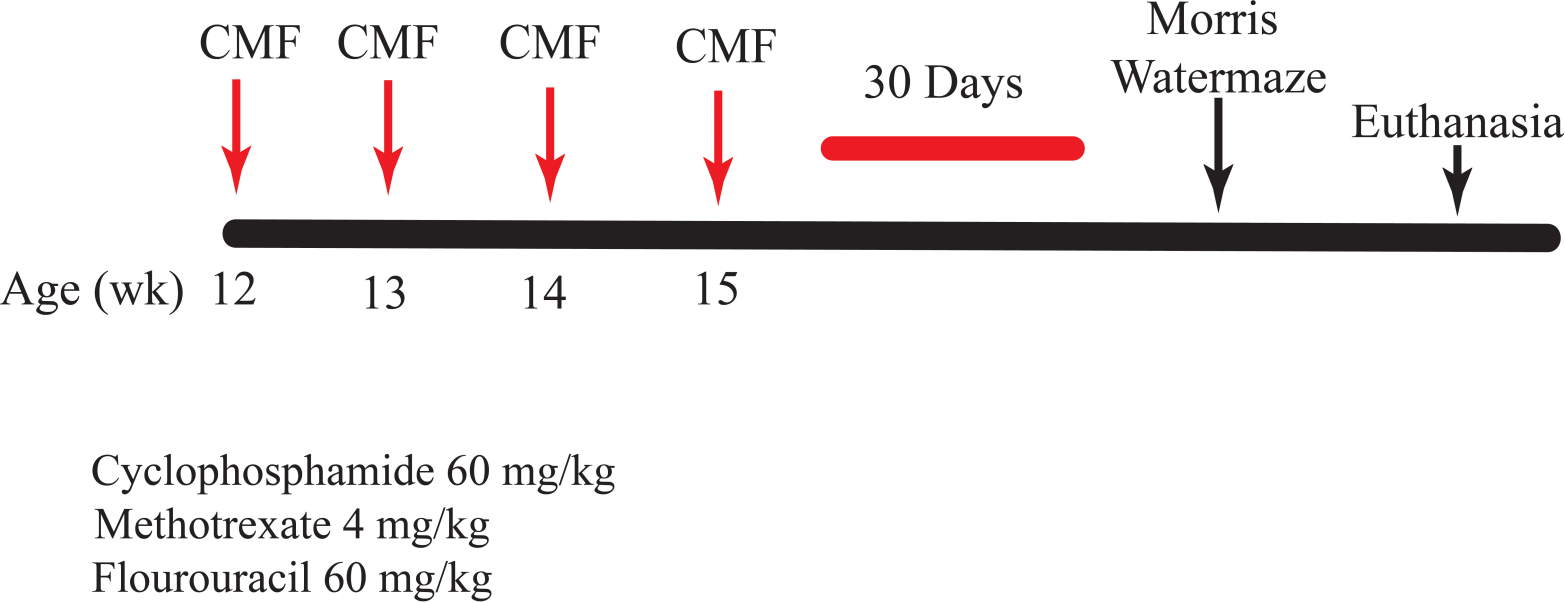
Study timeline. C57BL/6J mice, 12 weeks of age, were injected with CMF or saline. Behavior testing was initiated at 30 days following the final chemotherapy injection.

The treated group was administered a combination of CYP (60 mg/kg), MTX (4 mg/kg), and 5-FU (60 mg/kg) purchased from the UAMS Inpatient Pharmacy (Corley et al., 2023). The control group was administered saline (0.9% sodium chloride). CMF and saline were administered intraperitoneally weekly over 4 weeks, for a total of four injections (days 1, 8, 15, and 22). These dosages were determined by normalizing mouse body surface area. Drugs were diluted with sterile saline and then stored per the manufacturer’s instructions. Drugs were mixed immediately prior to injections. Behavioral testing was performed 30 days after the last injection. The mice were euthanized 30 min after the last probe trial, the hippocampal tissue was harvested, and fecal matter was collected, flash frozen in liquid nitrogen, and stored for analysis. The experimental timeline is presented in **Figure 1**.

### 2.3 Morris water maze

The Morris water maze, developed by Richard G. Morris in 1984, is considered a gold standard test in neuroscience and is used to test spatial learning and memory in murine models. In the test, the animal must rely on cues to learn the location of a submerged platform and escape the water. The maze consists of a circular pool (140 cm in diameter) filled with opaque water (24°C). Mice were trained to locate a visible platform. The testing schedule consisted of three phases: visible platform (days 1 and 2), hidden platform (days 3–5), and probe trials (end of days 3–5). The EthoVision XT video tracking system and software (Noldus Information Technology) was used to record the distance moved, the latency, and the average velocity of the animals in the visible platform, hidden platform, and probe trials. During the visible platform training, we measured the ability of the animal to learn a task. Each group was run through two of these sessions (spaced 2 h apart) per day for a total of four sessions. Each session consisted of three trials, with the start location and platform location moving to different quadrants with each trial. Each animal was released into a quadrant and trained to locate the platform with visual cues positioned around the pool. During the hidden platform training, we measured the acquisition of spatial learning. This trial was performed on days 3–5 in a manner consistent with the visible platform trial; however, the platform was submerged 1.5 cm below the water line. For both the visible and hidden platform trials, the test was terminated once the mouse found the platform. If the mouse was unable to find the platform, the technician would place their finger in the maze to guide the mouse to the platform. The mouse remained on the platform for 10 s. The probe trial was conducted 1 h after the last run on each day of the hidden platform trial. The mice were released in the quadrant opposite the target quadrant and were allowed to swim around the maze for 60 s. The amount of time spent in the quadrants was recorded and compared.

### 2.4 Proteomics

Total protein from each hippocampal tissue sample was reduced, alkylated, and purified by chloroform/methanol extraction prior to digestion with sequencing grade modified porcine trypsin (Promega). Tryptic peptides were labeled with tandem mass tag isobaric labeling reagents (Thermo Fisher Scientific) according to the manufacturer’s instructions and combined into one 16-plex TMTpro sample group. The labeled peptide multiplex was separated into 46 fractions on a 100 × 1.0 mm Acquity BEH C18 column (Waters) with an UltiMate 3000 UHPLC system (Thermo Fisher Scientific) with a 50-min gradient from 99:1 to 60:40 buffer A:B ratio under basic pH conditions (buffer A=0.1% formic acid, 0.5% acetonitrile; buffer B=0.1% formic acid, 99.9% acetonitrile; both buffers adjusted to pH 10 with ammonium hydroxide for offline separation) and then consolidated into 18 super-fractions. Each super-fraction was then further separated by reverse-phase XSelect CSH C18 2.5 μm resin (Waters) on an in-line 150 × 0.075 mm column with an UltiMate 3000 RSLCnano system (Thermo Fisher Scientific). Peptides were eluted with a 70-min gradient from 98:2 to 60:40 buffer A:B ratio. Eluted peptides were ionized by electrospray (2.4 kV) followed by mass spectrometric (MS) analysis on an Orbitrap Eclipse Tribrid mass spectrometer (Thermo Fisher Scientific) with multi-notch MS3 parameters. MS data were acquired with the Fourier transform mass spectrometry (FTMS) analyzer in top-speed profile mode at a resolution of 120,000 over a range of 375 to 1,500 m/z. Following collision-induced dissociation activation with a normalized collision energy of 35.0, MS/MS data were acquired with the ion trap analyzer in centroid mode and normal mass range. With synchronous precursor selection, up to 10 MS/MS precursors were selected for higher-energy collisional dissociation activation with a normalized collision energy of 65.0, followed by the acquisition of MS3 reporter ion data with the FTMS analyzer in profile mode at a resolution of 50,000 over a range of 100–500 m/z. Methods were performed by the UAMS Proteomic Core.

### 2.5 Western blotting

Samples of the frozen hippocampal tissues were homogenized with a Potter–Elvehjem mechanical compact stirrer, model number BDC2002 (Caframo LabSolutions), in a 1% Triton X-100 radioimmunoprecipitation assay (RIPA) buffer containing protease inhibitors (Sigma-Aldrich). The protein concentration was determined with a bicinchoninic acid (BCA) protein assay (Bio-Rad), and 30 µg of protein was added to a 2× Laemmli buffer containing β-mercaptoethanol (5%). Gel electrophoresis was performed, and the proteins were transferred to a nitrocellulose membrane. The membranes were incubated in rabbit anti-c-Fos (Abcam), mouse anti-Zif268 (Santa Cruz Biotechnology), mouse anti-Arc (Santa Cruz Biotechnology), or mouse anti-glyceraldehyde 3-phosphate dehydrogenase (GAPDH) (Santa Cruz Biotechnology) in TBS containing 0.1% Tween-20 and 5% nonfat dry milk at 4°C overnight. After incubating with horseradish peroxidase-conjugated goat anti-mouse or anti-rabbit IgG (Jackson Immunoresearch), the membranes were covered in enhanced chemiluminescence Plus Western Blotting Detection Reagent (GE Healthcare Life Sciences) and placed on a CL-Xposure Film (Thermo Fisher Scientific). The films were developed and imaged with an AlphaImager gel documentation system (ProteinSimple). Densitometry was performed with ImageJ software v.1.53 (National Institutes of Health). Antibodies were normalized to the loading control GAPDH and calculated relative to the expression of each target antibody in the saline controls.

### 2.6 Microbiome

Mouse fecal pellets were sent to RTL Genomics for DNA extraction, amplification, and sequencing of the V3–V4 small ribosomal subunit (16S rRNA) hypervariable region with the following primers: 5′-CCTACGGGNGGCWGCAG-3′ and 5′-GACTACHVGGGTATCTAATCC-3′ (Klindworth et al., 2013). MIMARKS (Yilmaz et al., 2011)-compliant sequencing data are available via the GenBank SRA under BioProject PRJNA1141459.

Microbiome analyses were performed with QIIME2 (version 2021.11) and demultiplexed, and primers were trimmed. FASTQ files were imported in QIIME2 as QIIME Zipped Artifacts (qza) with q2-import and visualized with q2-demux summarize via QIIME2. q2-cutadapt (CITE cutadapt) was used to trim primers from the paired-end reads (Martin, 2011). Amplicon sequence variants (ASVs)/exact sequence variants (ESVs) (Callahan et al., 2016) were generated from forward reads with DADA2 (Callahan et al., 2017) via q2-DADA2 plugin.

We used the q2-feature-classifier classify-sklearn plugin (Bokulich et al., 2018) and RESCRIPt (Robeson et al., 2021) to curate the SILVA NR99 v138.1 reference database for the V3–V4 hypervariable region. Taxonomy was assigned to ASVs with Naïve Bayes classifier trained on the SSU SILVA NR99 reference database (Pruesse et al., 2007; Quast et al., 2013). ASVs that were categorized as “Unclassified”, “Mitochondria”, “Chloroplast”, and “Eukaryotes”, and those not having at least phylum level classification were removed. The quality of the sequences was evaluated with q2-quality-control evaluate-seqs plugin by comparing the feature sequences to the curated SILVA reference; any sequences that did not have at least either a 90% identity or query coverage were removed. ASVs present with 10 reads or less and that appeared in less than two samples were removed. Data were rarefied at 8,500 reads per sample. Alpha-diversity metrics were estimated for observed taxa, Shannon Index, and Faith’s Phylogenetic Diversity (PD). Beta-diversity was estimated with UniFrac (weighted and unweighted) and Bray–Curtis dissimilarity and Jaccard with q2-diversity.

### 2.7 Villus height and crypt depth

We assessed the villus height and the crypt depth. Segments of proximal jejunum were obtained, fixed, and embedded so that four transverse sections were obtained per specimen, cut at 5 μm, and stained with hematoxylin and eosin (H&E). H&E-stained slides were used for villi length and crypt depth determination. Each stained section was examined for histopathological abnormalities on a microscope supported with a digital camera. Images were captured at 4× magnification for villus measurements and 10× magnification for crypt measurements. An average of 12 villi were analyzed for villous height and 12 crypts were analyzed for crypt depth measurements per animal at 20× magnification. The villus height was measured from the tip to the villus–crypt junction and the crypt depth from the base of the villus to the mucosa with ImageJ software v.1.53, *n*=5 per treatment group.

### 2.8 Statistical analysis

Data were expressed as means ± SD. Comparisons between means were carried out with mixed-effects repeated-measures analysis of variance (ANOVA) when analyzing the Morris water maze. Unpaired *t*-tests were performed for Western blot analysis. Statistical analysis was performed with Prism software version 9 (GraphPad); a probability level of less than 0.05 (*p* < 0.05) was accepted as statistically significant. Kruskal–Wallis and *post-hoc* tests were used for QIIME2 statistical analysis.

## 3 Results

### 3.1 Morris water maze

**Figure 2:**
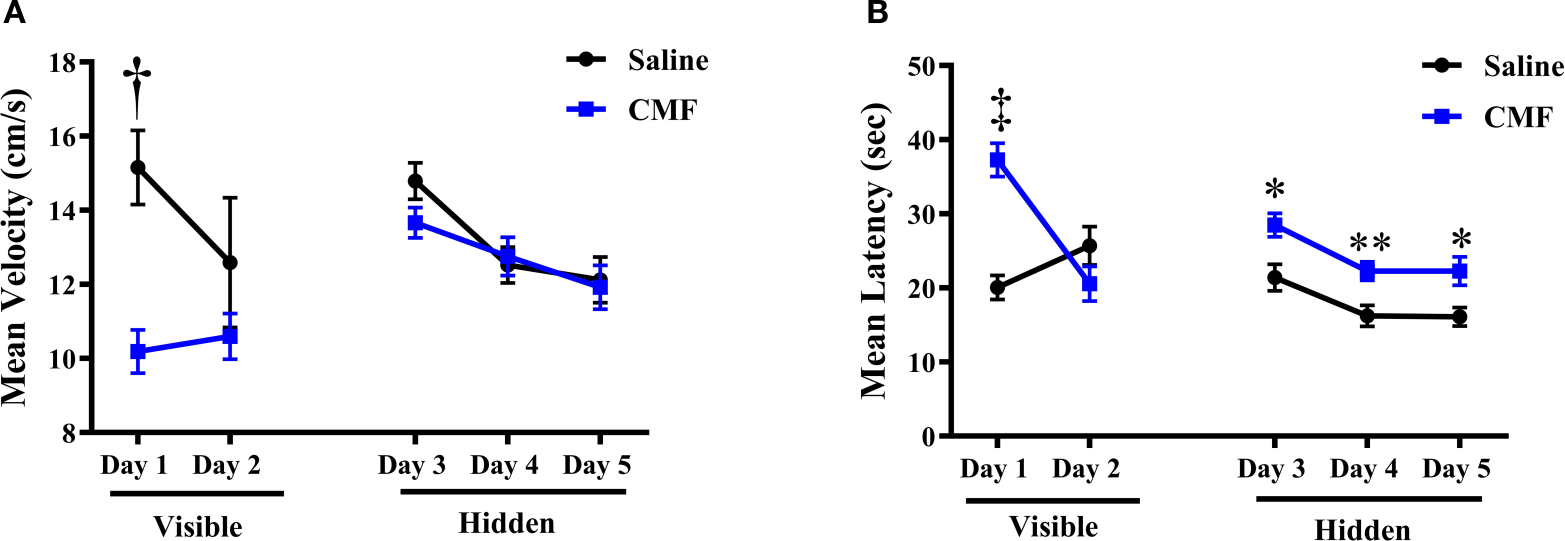
Velocity and latency measurements via the Morris water maze. **(A)** Visible (day 1 and 2) and hidden (days 3–5) platform analysis revealed on day 1, mice in the saline group swam significantly faster than the mice in the CMF group, but both groups showed a similar velocity throughout the reminder of testing. **(B)** During the visible-platform training, the CMF group latency was initially significantly greater than the saline group, but both groups decreased to similar values by day 2. When the platform was hidden, the saline group had a latency that was significantly less than the CMF group. Error bars represent the standard error of the mean (SEM). ‡*p*=0.0001; †*p* < 0.0001; *p=0.0142; **p=0.0098; *p=0.0227.

We assessed spatial learning and memory retention using the hidden/visual 5-day Morris water maze task. Mean velocity to the platform was assessed using a repeated-measures, mixed-model ANOVA. Results from this statistical analysis revealed that saline-treated mice swam significantly faster than CMF-treated mice in treatment-by-day interactions in the mean velocity [*F* (4, 92)=3.702, *p*=0.0077, **Figure 2A**]. Latency is characterized by the amount of time it takes the animal to reach the platform. Assessment of latency found that mice injected with saline exhibited significant decreases in mean latency in treatment-by-day interactions [*F* (4, 230)=9.015, *p* ≤ 0.0001, **Figure 2B**]. Another metric measured was distance moved. Both treatment groups swam similar distances to the platform and demonstrated significant treatment-by-day interactions [*F* (4, 230), *p*=0.0049] during visible platform training. Furthermore, saline- and CMF-treated mice displayed daily improvements in their ability to locate the targeted quadrant during hidden platform training (days 3–5).

**Figure 3:**
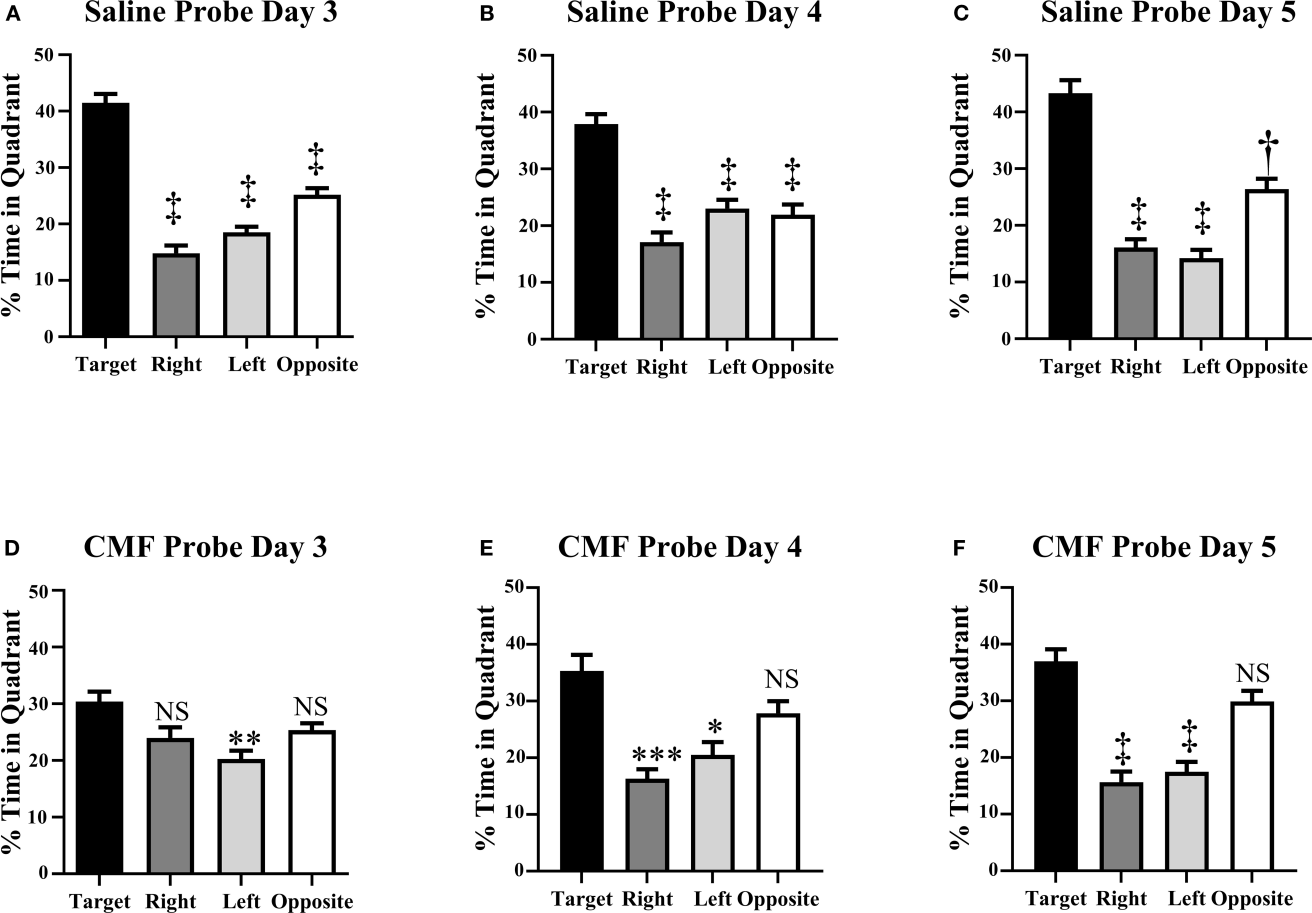
Spatial memory retention was tested during probe trials on days 3–5 of the Morris water maze. **(A–C)** Mice in the saline group spent significantly more time in the target quadrant than in the other quadrants. **(D–F)** Mice in the CMF group did not discriminate between the target, right, and left quadrant on days 3–5. Each bar represents the mean of 24 mice; error bars represent the SEM. ‡*p* < 0.0001; **p=0.0036; ***p=0.0002; *p=0.0181; †*p*=0.003; NS, not significant.

To assess spatial memory retention, probe trials were conducted on days 3–5 of the Morris water maze following the hidden platform trials, via removal of the platform. Every day of the probe trials, saline-treated mice exhibited significant preference for the target quadrant: day 3 [*F*_(2.353, 72.15)_, *p* < 0.0001; **Figure 3A**], day 4 [*F*_(2.565, 78.67)_=28.35, *p* < 0.0001; **Figure 3B**], and day 5 [*F*_(2.086, 63.97)_, *p* < 0.0001; **Figure 3C**]. Conversely, CMF-treated mice showed nearly no preference for the quadrants, only spending significantly less time in the left quadrant on day 3 [*F*_(2.307, 67.66)_, *p*=0.0012; **Figure 3D**]. On days 4 and 5, CMF-treated mice spent significantly less time in the left and right quadrants, although displaying no significant discrimination between the target quadrant and its opposing quadrant [*F*_(2.023, 59.33)_, *p* ≤ 0.0001 (**Figure 3E**) and *F*_(2.627, 80.57)_, *p* < 0.0001 (**Figure 3F**), respectively].

### 3.2 Proteomics

**Figure 4:**
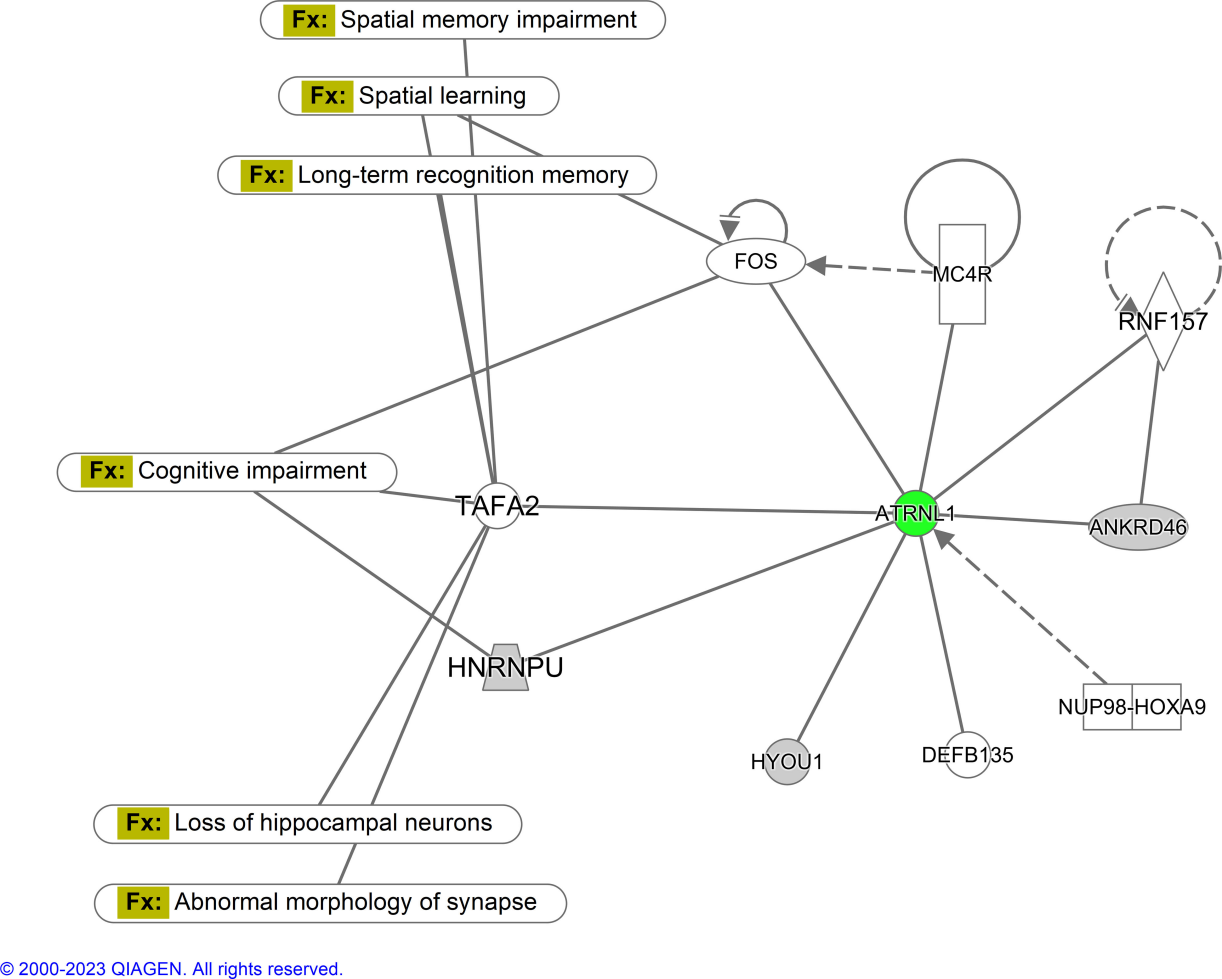
Ingenuity pathway analysis-generated network for hippocampal proteins. Parameters were set for a 1.5-fold change and a *p*-value < 0.05. The complete dataset contained 5,468 proteins; only 7 proteins fell within the criteria. In this diagram, green represents downregulated expression, and gray is either undefined or there is not enough information to be confident in determining its regulatory characteristics. Fx represents the function (*N*=6 per treatment).

Parameters were set for a 1.5-fold change and a *p*-value < 0.05. The complete dataset contained 5,468; only 7 proteins fell within criteria. In network 1, only one protein, Attractin like-1 (ATRNL1, a type 1 transmembrane protein), was predicted to be downregulated (**Figure 4**).

### 3.3 Western blotting analysis

**Figure 5:**
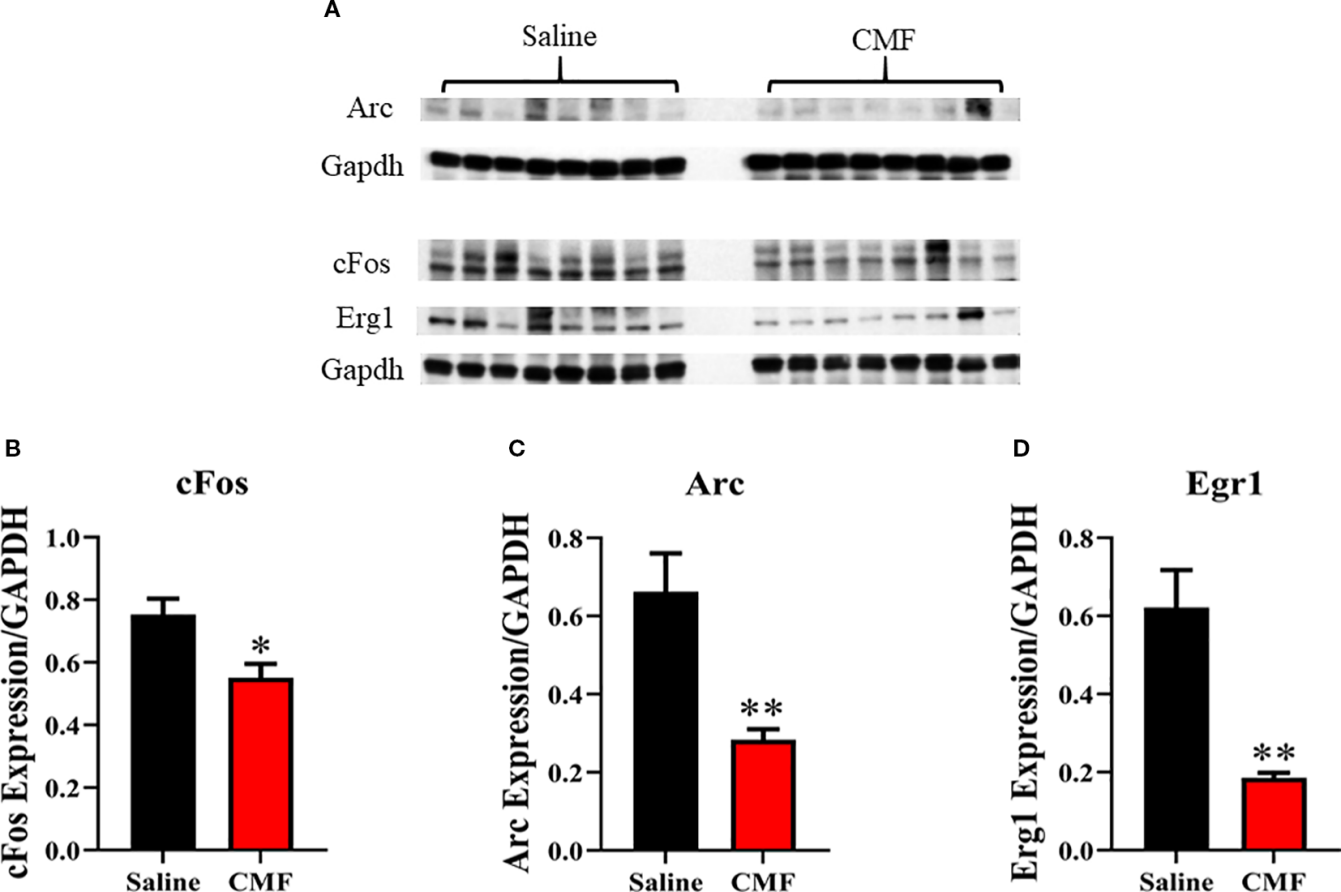
Immediate-early gene expression in the hippocampus. IEG expression was measured using Western blot analysis **(A)**. Protein expression was normalized to GAPDH **(B–D)**. Mice in the CMF group showed a significant reduction in IEG expression in the hippocampus. Average ± SEM, *N*=7–8. Bars represent SEM. *Denotes significant difference between saline- and CMF-treated mice. *p=0.0118; **p=0.0039; **p=0.0010.

Western blots are shown in **Figure 5A**. In this analysis, there was a significant decrease in the expression of c-Fos (*t*=2.925, *p*=0.0118; **Figure 5B**), Arc (*t*=3.502, *p*=0.0039; **Figure 5C**), and Erg1 (*t*=4.197, *p*=0.0010; **Figure 5D**) in the CMF group when compared to the saline group on a *t*-test.

### 3.4 Microbiome

**Figure 6:**
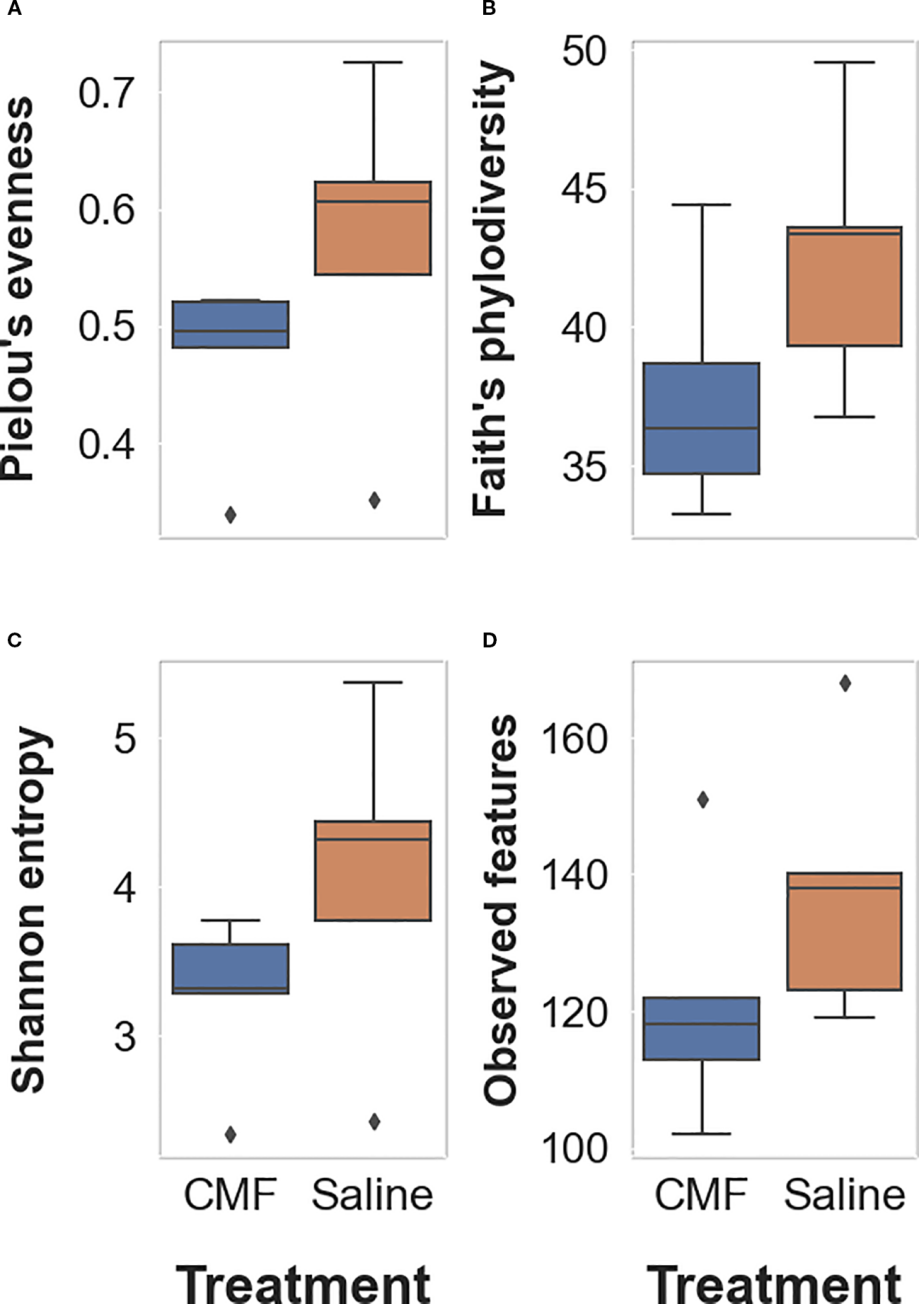
Alpha-diversity metrics. **(A)** Pielou’s metric measures evenness of the number of different species in each treatment group. **(B)** Faith’s phylodiversity measures the biodiversity that incorporates phylogenetic difference between species. **(C)** Shannon’s entropy is a measurement of the uncertainty of occurrence of certain events. **(D)** Observed features measure the number of observed events (e.g., species, variants, and genes) found within the group. The Kruskal–Wallis test was used to determine significance (*N*=5 per treatment).

Each alpha-diversity metric was measured for significance using the pairwise Kruskal–Wallis test, comparing the saline group to the CMF group. There were no significant differences identified among taxa within the CMF group compared to the saline group for Pielou’s evenness (**Figure 6A**; *p*=0.0758), Faith’s phylodiversity (**Figure 6B**; *p*=0.1172), Shannon entropy (**Figure 6C**; *p* ≥ 0.1172), and observed features (**Figure 6D**; *p*=0.1172).

**Figure 7:**
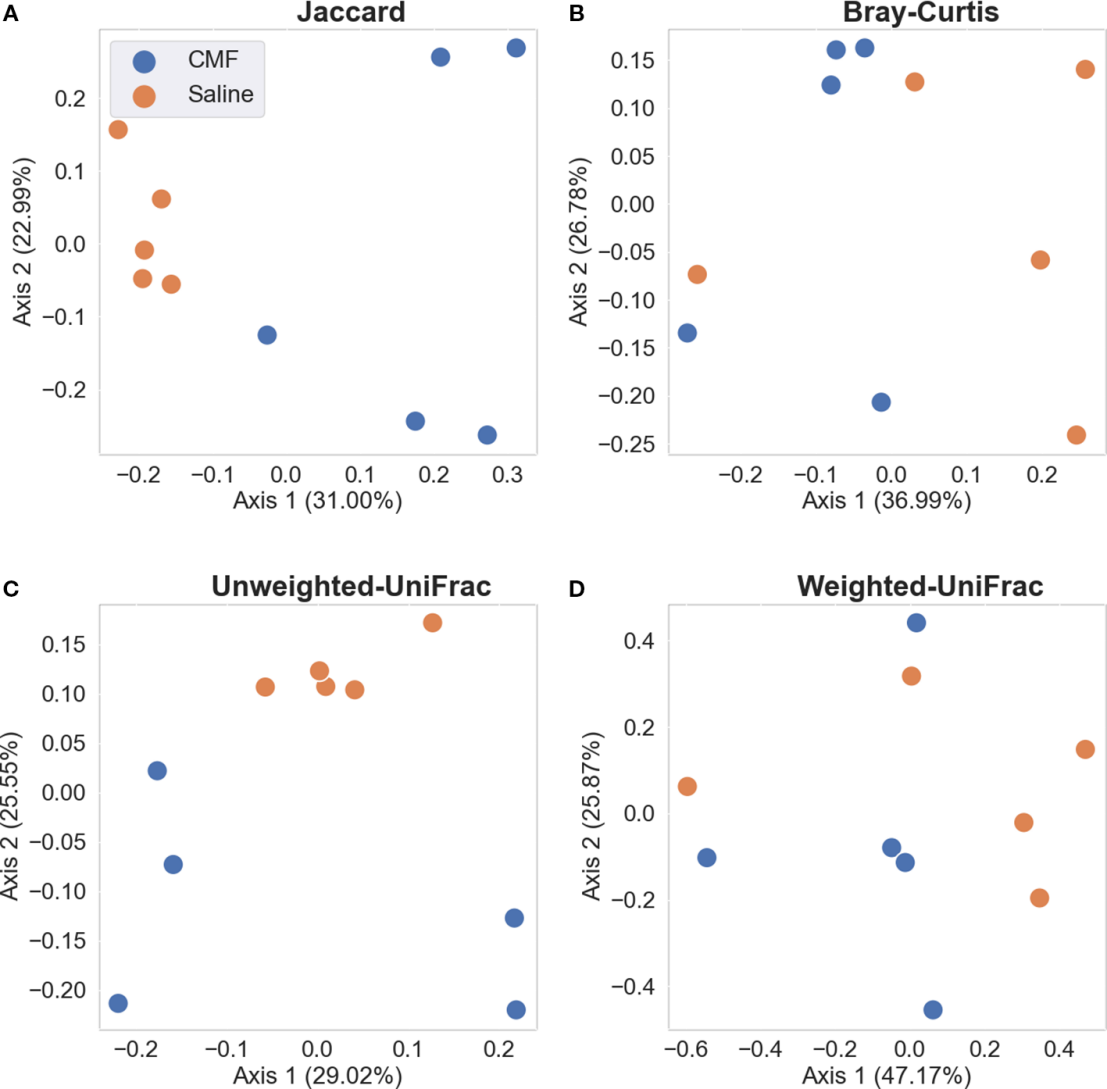
Beta-diversity metrics. **(A)** Jaccard and **(B)** Bray-Curtis, non-phylogenetic measures of richness and evenness, respectively. **(C)** Unweighted UniFrac and **(D)** Weighed UniFrac, phylogenetic measures of richness and evenness, respectively. Each dot represents a sample, blue represents saline-treated, and orange represents CMF-treated *N*=5 per treatment).

Beta-diversity was estimated with Jaccard distance (**Figure 7A**; *p* < 0.009), Bray–Curtis dissimilarity matrix (**Figure 7B**; *p*=0.12), Unweighted UniFrac distance (**Figure 7C**; *p* < 0.009), and Weighted UniFrac distance (**Figure 7D**; *p* =0.319) measurements. The quantitative-based metrics, i.e., Bray–Curtis and Weighted UniFrac, were not significant, whereas the qualitative metrics, Jaccard and Unweighted UniFrac, were significant.

### 3.5 Villus height and crypt depth

**Figure 8:**
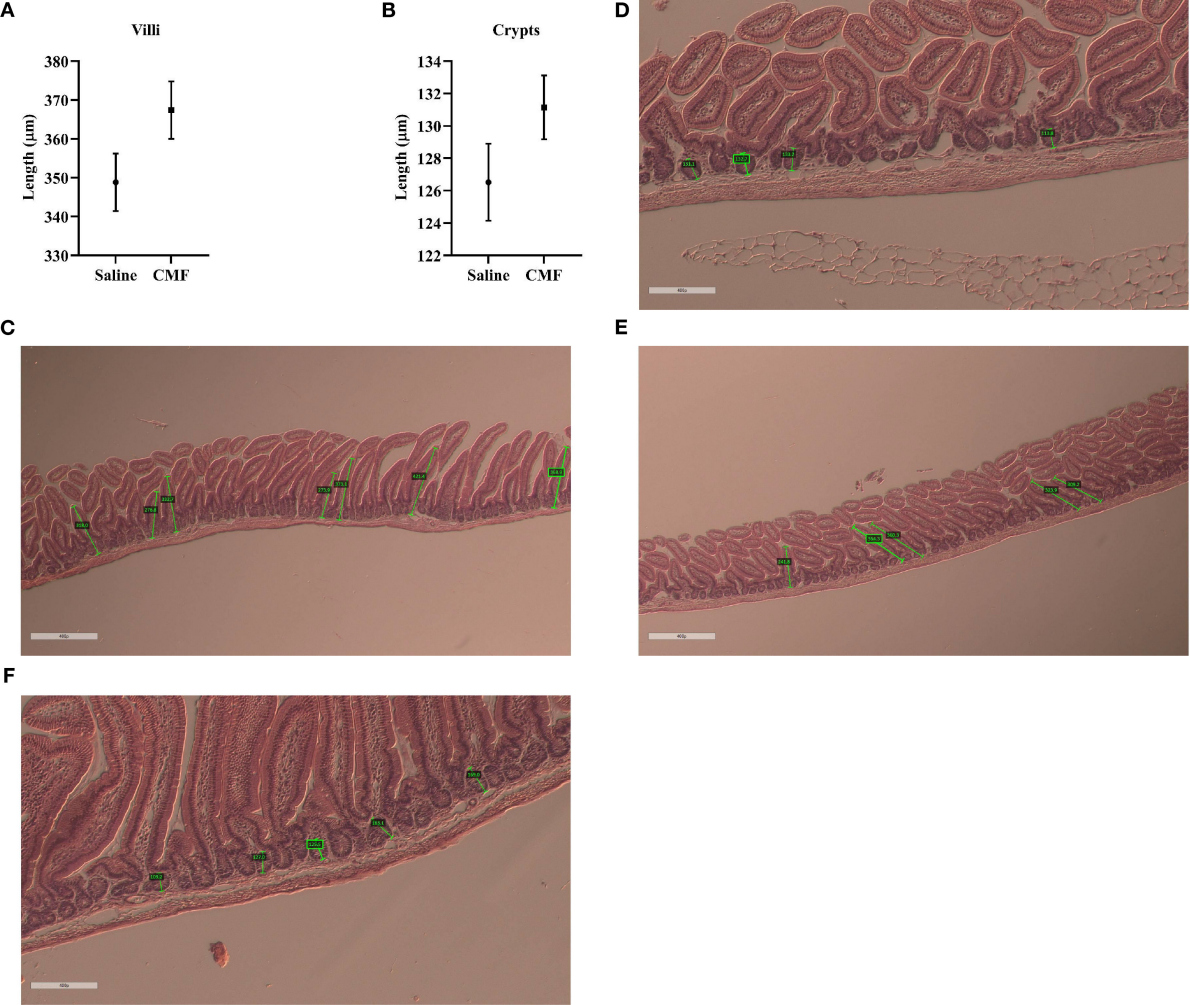
Changes seen 30 days after treatment. **(A, B)** Average villi length and crypt depth are not affected 30 days after treatment (*N*=5 per treatment). Histological images show a representative of **(C)** saline group villi, **(D)** CMF group villi, **(E)** saline group crypts, and **(F)** CMF group crypts (*N*=8 per treatment).

The intestinal derangement was not observed 30 days after CMF treatment. When the CMF group (348.8 μm) was compared to the saline group (367.6 μm), significance in average villi length was not observed (*t*=1.773, *p*=0.0785; **Figure 8A**). Crypt depth (**Figure 8B**) was not significantly shifted either (*t*=1.514, *p*=0.1324). Histological images show saline villi, CMF villi, saline crypts, and CMF.

## 4 Discussion

Our prior work has found that 2 weeks post-chemotherapy, 6-month-old female mice treated with CMF showed significant long-term memory impairments in the Morris water maze and a decrease in hippocampal arborization (Anderson et al., 2020). We have also shown that 24 h post-chemotherapy, 12-week-old female mice treated with CMF displayed depressive-like behaviors with deficits in social novelty in the Forced Swim and Three Chamber Sociability tests, respectively (Corley et al., 2023). Both studies use the same drug doses and injection paradigm as presented herein. This study was designed to establish if cognitive impairments still occur 30 days post-CMF. We tested spatial memory using the Morris water maze task and found that CMF-treated mice exhibited an inability to retain memory in the probe trials. Our proteomics analysis identified the protein Attractin-like 1 (ATRNL-1), which was found to have an undefined connection with three proteins related to cognitive impairments, spatial learning, and memory deficits. When probing for immediate-early genes (IEGs) via Western blots, a significant decrease in c-Fos, Zif268, and Arc expression was observed in the CMF treatment group. Lastly, there were no notable changes in gut morphology between treatment groups, mirroring the lack of detectable differences in microbial diversity between the treatment groups.

The Morris water maze is a spatial learning task that is hippocampal-dependent (Morris et al., 1982). The hippocampus has been vastly studied and shown to be critical for long-term episodic memory (Bird and Burgess, 2008; Lisman et al., 2017). Further research has shown that the anatomical formation of the hippocampus supports its role in mediating spatial cognition and memory (Chiba et al., 1994; Fortin et al., 2002; Bird and Burgess, 2008; Hartley et al., 2014; Lisman et al., 2017). Both CMF- and saline-treated groups demonstrated the ability to learn during the Morris water maze. Notably, however, the saline group on day 1 swam significantly faster to the platform than the CMF group measured via mean velocity. Days 3–5 of testing revealed that CMF-treated mice were unable to distinguish between the target and non-target quadrant, conveying a lack of memory retention. This observation follows similar findings to that of several studies. A study conducted by Kinra et al. reported that mice administered CMF once a week for 3 weeks displayed significantly lower retention time and higher latency to platform in the Morris water maze (Kinra et al., 2021). Anderson et al. showed in the Morris water maze that CMF-treated mice could not distinguish between the target and non-target quadrants on days 3 and 4 (Anderson et al., 2020). As monotherapies or doublets, CYP, MTX, and 5-FU have been shown to affect spatial cognition as well. Mishra et al. found that mice treated with a single dose of CYP were highly immobile and had higher latency to platform during the Morris water maze compared to control mice (Mishra et al., 2022). Mice given MTX (250 mg/kg) showed longer latency time to the platform during probe trials compared to control mice, despite showing the ability to learn (Seigers et al., 2008). A combination of 5-FU and MTX was administered to 2-month-old female BALB/C mice and were then tested in the water maze 1 week after treatment. 5-FU+MTX-treated mice were more latent to the platform and made more errors in an attempt to find the target than control mice on days 1 and 2, showing that learning was initially impaired (Winocur et al., 2006). Interestingly, on day 5, our study shows that CMF-treated mice indicated improved memory retention by spending significantly more time in the target quadrant than in non-target quadrants than on previous days.

Using QIAGEN Ingenuity Pathway Analysis (IPA), a network of proteins was rendered to depict the relationship between dysregulated proteins found within our study. The network compared the CMF group to the saline group. ATRNL 1 is a transmembrane protein that is thought to be integral to membrane integrity (Walker et al., 2007) and was the only protein predicted to be downregulated. This protein was shown to have an ambiguous relationship with proteins c-Fos, TAFA2, and HNRNPU. c-Fos, a proto-oncogene and one of the most extensively studied IEGs (Herrera and Robertson, 1996), encodes a protein rapidly upregulated in response to membrane depolarization and voltage-gated calcium influx, enabling neural action potentials. Beyond its role as a marker of neuronal activation, c-Fos has been implicated in learning, memory consolidation, and synaptic plasticity, particularly in spatial memory circuits. For example, female rats trained in acrobatic conditioning displayed a significant increase in c-Fos-positive cells within the motor cortex compared to control and inactive animals, underscoring its role in motor learning (Kleim et al., 1996). Méndez-Couz et al. found an increase in c-Fos protein expression in the amygdala following spatial memory extinction from the Morris water maze (Mendez-Couz et al., 2014). c-Fos knockout mice have been shown to exhibit impairment in hippocampal-dependent spatial memory and long-term potentiation (LTP). Fleischmann et al. showed that c-Fos knockout mice could not discriminate between target and non-target quadrants during the probe trial of the Morris water maze. Moreover, there was a decrease in LTP in hippocampal synapses in the CA3–CA1 subregions (Fleischmann et al., 2003).

HNRNPU encodes for the heterogeneous nuclear ribonucleoprotein U and is critical for RNA splicing as well as chromatin organization (Sapir et al., 2022). HNRNPU has been found to be important for development. For example, an HNRNPU mutation within mice resulted in embryonic growth retardation and early mortality (Roshon and Ruley, 2005). Sapir et al. investigated the role of HNRNPU in brain development. Their work revealed that deletions to HNRNPU resulted in the splicing of genes involved in neuronal survival and synaptic formation. This study also supported the findings of Roshon and Ruley, discovering that the developing mouse brain expresses high levels of HNRNPU in the cortical plate and mitotic cells (Sapir et al., 2022). TAFA2 is a central nervous system (CNS)-specific cytokine that aids in CNS regulation (Liang et al., 2023). Wang et al. found that TAFA2 knockout mice showed increased anxiety-like behaviors in the open field test and elevated plus maze. The study also used the Morris water maze and novel object recognition paradigm to assess spatial, short- and long-term memory—finding significant deficits in each (Wang et al., 2018). Another study has suggested that TAFA2 is vital for neuronal cell survival via binding to the ADGRL1 protein to activate the cAMP-PKA-CREB-BCL2 signaling pathway and prevent apoptosis (Liang et al., 2023). Consequently, we thought it important to probe similar IEGs in hopes of deepening our mechanistic understanding of the behavioral results from the hippocampal-dependent, spatial learning task of the Morris water maze on a neuronal level. Arc and Zif268 were ideal candidates to do so.

Brain IEGs are a class of genes that are rapidly and transiently activated by neuronal activity (Abraham et al., 1991). IEGs can be further divided into two functional classes: regulatory or effector. Regulatory IEGs encode proteins that indirectly affect neural physiology by increasing or decreasing “downstream” gene expression. Conversely, effector IEGs encode proteins that have a more defined role at the synapse (Guzowski et al., 2001; Davis et al., 2003). Both classes of IEGs are necessary in supporting the mechanisms that underlie neuronal plasticity such as LTP, kindling, cellular regeneration, and learning. In particular, Arc is an effector IEG. Arc, activity-regulated cytoskeleton-associated protein, is thought to be essential for memory consolidation and contributes to synaptic plasticity via α-amino-3-hydroxy-5-methyl-4-isoxazolepropionic receptor (AMPAR) regulation. AMPARs are dynamic ionotropic glutamate receptors that mediate most of the excitatory synaptic transmission in the brain (Diering and Huganir, 2018). As an effector IEG, the mRNA of Arc can be localized to post-synaptic dendrites via cytoplasm permeation after action potential prompted by behavior (Minatohara et al., 2015). It is a plasticity protein. Zif268, like c-Fos, is a regulatory IEG. Part of the early growth response (Egr) family, Zif268 is a transcription factor that controls gene expression—aiding in cell development and function (Veyrac et al., 2014). Furthermore, it is believed that the Egr genes serve to connect neurotransmitter action with altered gene expression. Zif268 is highly expressed in the hippocampus, particularly in the CA1 region. Robert et al. found that there was a significant increase in Zif268 levels in the hippocampal CA1 following the induction of LTP in rats compared to control animals (Roberts et al., 1996). In another study, rats were conditioned using the Avoidance Shuttle Box training paradigm. An increase in Zif268 mRNA was subsequently observed (Nikolaev et al., 1992). A couple of studies have presented congruent results. Briones and Woods showed that CMF had deleterious effects on hippocampal cell proliferation as well as spatial and long-term memory in the Morris water maze task (Briones and Woods, 2011). Anderson et al. observed long-term memory deficits in CMF-treated mice. Further investigation revealed that CMF reduced hippocampal dentate gyrus dendritic length and mushroom spines. Importantly, mushroom spines are considered “memory” spines (Bourne and Harris, 2007). These studies provide context to our findings. Our results are consistent with prior and current research, supporting the notion that CMF deteriorates cognition as well as learning and memory function on the neural level.

Emerging research over the last decade has identified the microbiome as a contributing factor to outcomes for neurological changes (Luczynski et al., 2016; Jordan et al., 2018; Grant et al., 2021). Gastrointestinal mucositis (GIM), an inflammation of the intestinal mucosa, is an adverse side effect that can be induced by chemotherapy. GIM has been shown to decrease quality of life, decrease survivorship, and increase the commodity rate of patients with cancer (Sougiannis et al., 2021). Women are at higher risk for developing GIM, as well as patients treated with 5-FU mono- or polytherapy (Basile et al., 2019). MTX can also induce GIM (Higuchi et al., 2020). In fact, one of the most common adverse effects of MTX is gastrointestinal toxicity (Zhou et al., 2018). To further characterize CMF chemotherapy, we evaluated the alpha- and beta-diversity of the microbiome 30 days post-treatment. Differences in alpha-diversity between the CMF and saline groups were not observed (**Figures 7**, **8**). We analyzed beta-diversity quantitatively (Bray–Curtis and Weighted UniFrac) and qualitatively (Jaccard and Unweighted UniFrac) (Lozupone et al., 2007). Quantitative analyses revealed no distinct separation or clustering between CMF and the saline treatment group, meaning that the two treatment groups share many of the same phylogenetically related and abundant taxa. Qualitative beta-diversity analysis revealed that the two treatment groups were significantly different, indicating that the microbial communities of these two treatment groups still share many low abundant taxa after 1 month. The reduction of altered microbial communities presented here after 1 month is in stark contrast to that observed after 24 h, in which the CMF and saline-treated mice differed significantly in both alpha- and beta-diversity (Corley et al., 2023). These vastly different results may suggest that the microbiome, in the absence of CMF, displayed resilience and was able to return to a presumed “near-healthy” state after 1 month. Notably, the study at present only evaluated microbiome alpha- and beta-diversity 30 days following chemotherapeutic treatment. A limitation of this study is the absence of reagent blanks and Zymo/ATCC mock communities as positive controls, which may limit the ability to distinguish true biological signals from potential contamination or sequencing artifacts. Future studies will address this by including an aliquot of mock community DNA and either a reagent solution blank or PCR template blank as a negative control for sequencing, with all controls processed in triplicate.

Comparable studies have used shorter timelines—typically ranging from 1 to 2 weeks post-treatment. Lu et al. showed that following five daily doses of 5-FU, 5-FU GIM-induced mice had lower ACE, Chao, and Shannon indices than control (Lu et al., 2022). A similar result was presented by Li et al., such that rats injected with 5-FU for 3 days displayed a decrease in alpha- and beta-diversity as measured by Unweighted UniFrac principal coordinates analysis (PCoA) as well as Chao and Shannon indices (Li et al., 2017). Another study using MTX found that rats treated with MTX every 3 days for 7 or 14 days showed a significant decrease in alpha-diversity compared to control (Zhou et al., 2018). Chao1, ACE, Observed species, and Shannon indices were used for analysis. Recently, Chen et al. showed rats that initially received MTX via intrathecal injection and then via intraperitoneal injection once a week for 2 weeks had a significant decrease in beta gut microbiota diversity as measured by Unweighted UniFrac (Chen et al., 2024). Interesting, Chao1 and Shannon indices did not reveal any significant difference in alpha-diversity. A study conducted by Chen, Sun et al. administered 80 mg/kg of CYP to saline-treated mice after 10 days. After receiving CYP on days 10, 12, and 14, animals were sacrificed and tissues were collected; Chao1 and ACE indices showed that CYP-treated mice had lower alpha-diversity than control in the cecum (Chen et al., 2021). Additional research using multiple time points of analysis may prove useful in unraveling the complex underpinnings of the microbiome brain axis in tandem with the tight interactions of host gut morphology and microbial communities. Research on the long-term effects of chemotherapy on the MGB remains limited. Growing interest in this realm of cancer research is promising and is essential to identifying how the microbiome may facilitate long-term chemobrain and the amelioration of its side effects.

Notably, taxanes and alkylating agents, such as the adjuvants that compose CMF, are well known to induce chemotherapeutic-GIM (Akbarali et al., 2022). Various studies have shown that administration of CTX, MTX, and 5-FU poly- and monotherapies yields an increase in interleukin-1β (IL-1β) expression in response to chemotherapeutic-induced GIM (Wu et al., 2011; Xiang et al., 2011; Yucel et al., 2016). IL-1β is a pro-inflammatory cytokine that is critical to immune system regulation. Evidence has also suggested that IL-1β is a neuromodulator required for healthy brain activity (Schneider et al., 1998). Interestingly, some research proposes that sustained expression of IL-1β may have an antagonistic relationship with hippocampal neurons. Utilizing the transgenic mouse model IL-1β^XAT^ (Shaftel et al., 2007) for hippocampal IL-1β overexpression, work by Moore et al. has shown that IL-1β^XAT^ mice displayed spatial memory retention deficits in the Morris water maze task following 2 weeks of IL overexpression (Moore et al., 2009). Both male and female IL-1β^XAT^ mice exhibited significantly longer path lengths to the platform than control mice. Using the same time frame, Hein et al. demonstrated similar results and found that male and female IL-1β^XAT^ mice spent significantly more time in non-target quadrants during probe trials of the Morris water maze (Hein et al., 2010). Hein et al. also showed that in unconditioned IL-1β^XAT^ mice, there was a significant decrease in basal Arc mRNA expression compared to control. Surprisingly, contextual conditioning did not significantly increase basal Arc mRNA expression in IL-1β^XAT^ mice, regardless of gender. These studies may provide a different lens of interpreting our results. It could be that IL-1β expression was upregulated and potentially overexpressed due to the CMF chemotherapeutic regimen, which was shown to be possible by previous studies (Briones and Woods, 2014). The increase in IL-1β expression may also explain the difference in beta-diversity, such that Wu et al. have shown that IL-1β can impact commensal microbiota in mice (Wu et al., 2022). Moreover, the possibility of IL-1β overexpression may have persisted during the Morris water maze task, which may provide an ancillary reason for the observed cognitive deficits. Subsequent studies could aim to elucidate the mechanistic relationship between the MGB and pro-inflammatory cytokines, with particular emphasis on IL-1β and its antagonist IL-1Ra.

## 5 Conclusion

This research provides novel insights into what deficits occur after an extended time frame post-treatment with combination CYP, MTX, and 5-FU chemotherapy. The present study shows that treatment with CMF induced impairments in spatial memory and reduced c-Fos, Arc, and Zif268 expression without producing many protein changes in the hippocampus. Our study emphasizes the capability of CMF to engender persistent hippocampal-dependent cognitive dysfunction, represented by lack of memory retention in the Morris water maze probe trials 30 days post-treatment. These findings are novel and have not been demonstrated in previous CMF studies because of their shorter timelines. Additionally, our results suggest that c-Fos, Arc, and Zif268 may play a significant role in these observed cognitive deficits. Modulation in IEG expression in a female mice chemobrain model in the future could provide novel insights to further investigate the relationship between chemotherapy, IEGs, and cognition. An abundance of research has shown that our targeted IEGs contribute, directly or indirectly, to synaptic plasticity and stabilization of excitatory synapses at major information hubs in the brain. The reduction of these IEGs may explain the continual cognitive impairments seen in this study. Finally, we observed no changes in alpha-diversity and limited significant changes qualitative beta-diversity. Given the lack of differences in gut morphology between the treatment groups, it is not surprising that there were limited differences observed in overall microbial community structure between the treatment groups, indicating that the microbial community structure is following improvements in gut health and morphology. One limitation of our study is that we only examine one time point, which only provides a snapshot into the cognitive changes. Future studies should consider utilizing multiple time points of analysis. Given the varying results of similar studies, investigating the short- and long-term effects of chemotherapy in parallel is perhaps paramount in uncovering the underlying mechanistic relationships within the MGB axis. In addition, future studies incorporating immunostaining for tight junction markers or cytokine profiling could more precisely assess gut barrier integrity and its relationship to the observed behavioral and molecular outcomes. This is particularly true for women in LMICs who do not have the same access to modern breast cancer chemotherapeutic treatments as their Western counterparts. Continued research on CMF chemotherapy is imperative for improving the knowledge, survivorship, and quality of life for breast cancer survivors across the globe.

## Data Availability

The datasets presented in this study are available from the NCBI-SRA (BioProject PRJNA1141459). Proteomic data is available through PRIDE (Accession PXD063819). Further inquiries can be directed to the corresponding authors.
